# High-Performance Two-Dimensional InSe Field-Effect Transistors with Novel Sandwiched Ohmic Contact for Sub-10 nm Nodes: a Theoretical Study

**DOI:** 10.1186/s11671-019-3106-8

**Published:** 2019-08-15

**Authors:** Jiaduo Zhu, Jing Ning, Dong Wang, Jincheng Zhang, Lixin Guo, Yue Hao

**Affiliations:** 1 Wide Bandgap Semiconductor Technology Disciplines State Key Laboratory, School of Microelectronics, Xidian University, Xi’an, 710071 China; 2 Shaanxi Joint Laboratory of Graphene, Xidian University, Xi’an, 710071 China; 3 School of Physics and Optoelectronic Engineering, Xidian University, Xi’an, 710071 China

**Keywords:** InSe, Field-effect transistor, Density functional theory, Non-equilibrium Green function, Ohmic contact

## Abstract

Two-dimensional (2D) InSe-based field effect transistor (FET) has shown remarkable carrier mobility and high on-off ratio in experimental reports. Theoretical investigations also predicated the high performance can be well preserved at sub-10 nm nodes in the ballistic limit. However, both experimental experience and theoretical calculations pointed out achieving high-quality ohmic has become the main limiting factor for high-performance 2D FET. In this work, we proposed a new sandwiched ohmic contact with indium for InSe FET and comprehensively evaluated its performance from views of material and device based on ab initio methods. The material properties denote that all of fundamental issues of ohmic contact including tunneling barrier, the Schottky barrier, and effective doping are well concerned by introducing the sandwiched structure, and excellent contact resistance was achieved. At device performance level, devices with gate length of 7, 5, and 3 nm were investigated. All metrics of sandwiched contacted devices far exceed requirement of the International Technology Roadmap for Semiconductors (ITRS) and exhibit obvious promotion as compared to conventional structures. Maximum boost of current with 69.4%, 50%, and 49% are achieved for devices with 7, 5, and 3 nm gate length, respectively. Meanwhile, maximum reduction of the intrinsic delay with 20.4%, 16.7%, and 18.9% are attained. Moreover, a benchmark of energy-delay product (EDP) against other 2D FETs is presented. All InSe FETs with sandwiched ohmic contact surpass MoS_2_ FETs as well as requirement from ITRS 2024. The best result approaches the upper limit of ideal BP FET, denoting superior preponderance of sandwiched structures for InSe FETs in the next generation of complementary metal-oxide semiconductor (CMOS) technology.

## Introduction

Two-dimensional (2D) semiconductors have attracted much interest in electronic devices due to their appealing applications for the next generation of complementary metal-oxide semiconductor (CMOS) technology [1, 2]. Their ultra-thin thickness and good dielectric property can provide excellent electrostatic gate control to suppress the well-known short channel effects [3]. In addition, as few layers of 2D materials usually possess smooth surface with lack of dangle bonds, superiority carrier mobility of 2D materials can be well preserved in ultrathin body systems as compared to conventional semiconductor [4]. Except for the gapless graphene, most of synthesized 2D semiconductors like transition metal dichalcogenides (TMDs), black phosphorus (BP), and indium selenide (InSe) possess none-zero band gap and are demonstrated to be suitable for field-effect transistor (FET). TMDs-based FETs have shown high on-off ratio as much as 10^8^ and low leakage current in short channel devices, benefitting from the heavy effective mass [5]. BP-based FETs have presented outstanding current and switching characteristic [6], due to the high mobility of ~ 1000 cm^2^/V s and anisotropic transport property [7]. Recently, InSe was demonstrated to present a superiority mobility of ~ 2000 cm^2^/V s at room temperature [8, 9], and FET based on InSe revealed a high on-off ratio of 10^8^ [10]. First-principle calculations also identified that InSe FET can be well scaled down to sub-10 nm in the ballistic limit [11, 12]. However, due to the neglect of contact resistance and hypothesis of heavily doping, approaching the theoretical limit is still challenging in real applications. In fact, as reliable doping method and way to high-quality ohmic contact are still lacking, FETs based on 2D materials including InSe are usually Schottky barrier (SB) FET [13–16]. The SB at the active regions yields large contact resistance, and low doping level further degrades current density. Achieving low contact resistance with sufficiently doped active regions has become the main limiting factor for 2D materials-based FET (2D FET) to achieve high performance [17–19].

Aiming at above issues, we proposed a novel sandwiched ohmic contact for InSe FET. Indium was selected as the electrode metal, as recent experimental and theoretical studies suggest that indium can be a promising candidate for InSe FET to achieve good performance [20–22]. We theoretically evaluated the ohmic contact quality and performance of devices with gate length of 7, 5, and 3 nm following the framework of the International Technology Roadmap for Semiconductors 2013 (ITRS) [23]. It should be noted although ITRS has been replaced by the International Roadmap for Devices and Systems (IRDS) [24], ITRS2013 presents a clear scaling trend for transistor and has been still adopted in recent studies [25, 26]. This manuscript is arranged as follows: first, electrical properties of sandwiched and conventional (top) contacts are investigated. Second, device performance metrics such as on-state current and intrinsic delay are evaluated and compared with requirements of ITRS. Finally, benchmark of power-delay product versus intrinsic delay is presented to compare against other 2D materials-based devices.

## Methods

**Fig. 1:**
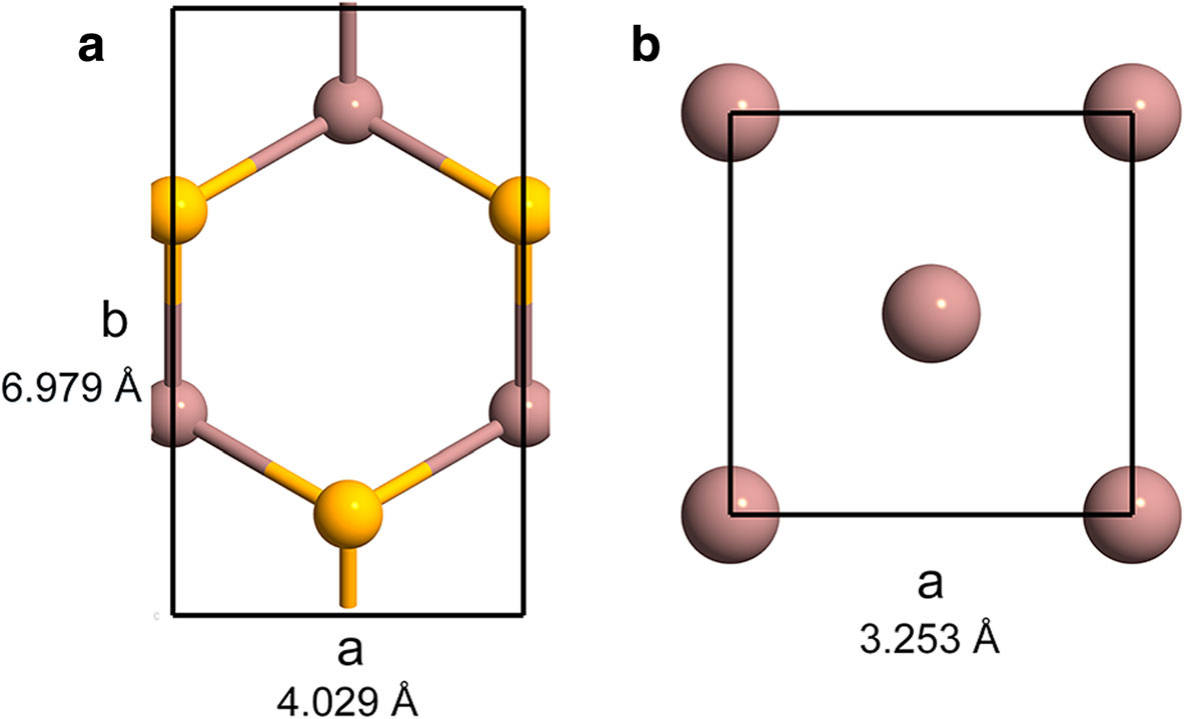
Top view of unit cell for InSe (**a**) and indium (**b**), respectively

All of atomic structures were optimized by VASP [27]; energy cut of 335 eV was employed during all calculations. Unit cell of InSe was relaxed with stress criterion of 0.01 eV/Å under the framework of MetaGGA of SCAN [28]. Lattice parameters of metal indium were obtained from handbook of chemistry and physics [29]. As shown in Fig. 1, the lattice constant of InSe is 4.029 Å, which is in very good agreement with experimental reports [30, 31].

**Fig. 2:**
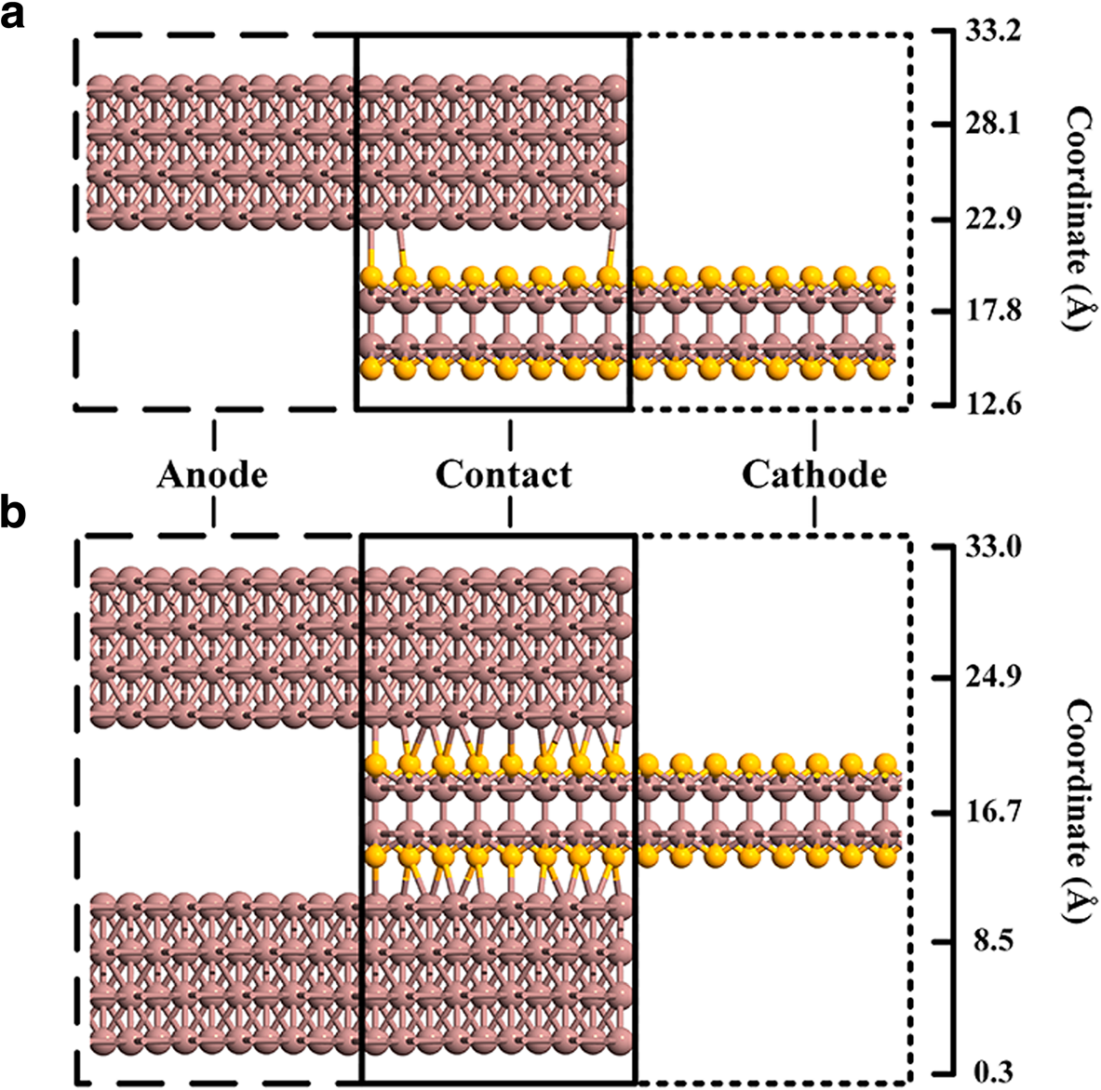
Atomic structures of contact and related two-probe device used for evaluation of contact resistance. **a**, **b** are for top and sandwiched contacts, respectively_._ The coordinates denote the location of atoms in the out-plane direction

The initial structure of indium on InSe was built with 4 × 1 × 1 and 5 × 2 × 1 unit cells of InSe and indium (001) surface, respectively. The mean absolute strain was 1.32%, which is sufficient to preserve the intrinsic properties of the material. As shown in Fig. 2a, b, the sandwiched structure was built with indium/InSe/indium layers, indium of bottom and top sides has mirror symmetry with center of InSe. Both of hybrid structures were relaxed with van der Waals (vdW) functional of optb88 with criterion of force on each atom lower than 0.02 eV/Å [32, 33]. The finnal contact area is 16.19 Å × 6.41 Å. The resistance of ohmic contact was then evaluated by a two-probe device as shown in Fig. 2a, b. Getting rid of unnecessary resistance from semiconductor out of contact regions, InSe in the cathode was heavily doped with 1 × 10^14^ e/cm^2^ for both top and sandwiched contacts.

**Fig. 3:**
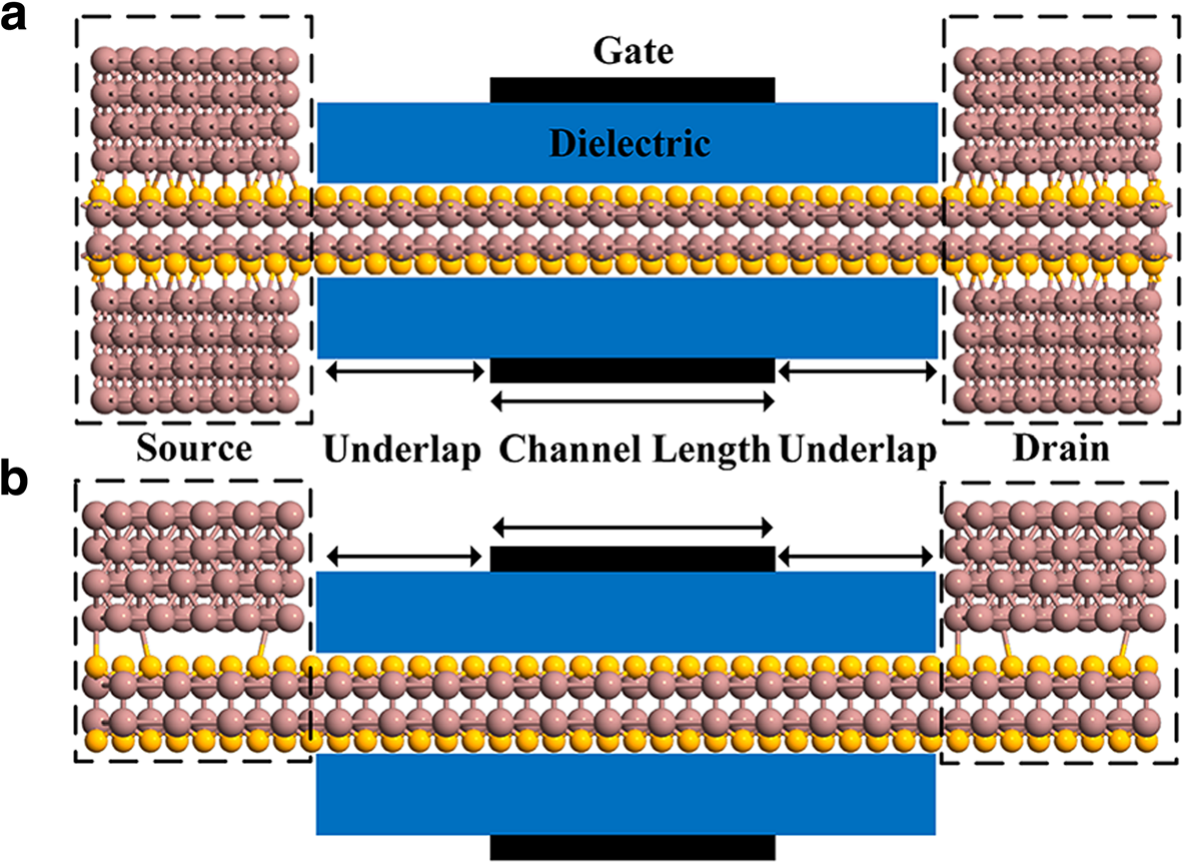
Geometries of InSe FETs with sandwiched (**a**) and top (**b**) contacts

As for the evaluation of device performance, geometry of InSe FET with sandwiched and top ohmic contacts is shown in Fig. 3a, b, respectively. All devices and nodes name follow requirement from ITRS and IRDS, respectively. Device parameters are listed in Table 1. To suppress the intra-band tunneling, 1 nm underlap (UL) was applied at gate length of 3 nm. In contrary to ohmic contact modeling, none of parts in devices was intentional doped. The devices were built by merging the source, drain, and channel along transport direction. The channel and its two interfaces with active regions were additionally relaxed with fixed source and drain. All simulations were based on non-equilibrium Green’s function (NEGF) theory and carried out by QuantumATK with fully self-consistent calculation [34–36], which was usually employed to design and investigate transistors at sub-10 nm nodes [17, 37–39]. Double-zeta polarized basis set were employed with mech-cut off of 90 Rydberg. Monkhorst pack k-point mesh was sampled with density of 8/Å^−1^ × 11/Å^−1^ × 180/Å^−1^. Parallel conjugate gradient solver is chosen as the Poisson solver for the sake of efficiency. The current of all devices can be then obtained by solving the Landauer-Büttiker formula [40]:

$$ I\left({V}_{\mathrm{Bias}}\right)=\frac{2e}{h}\int T\left(E,{V}_L,{V}_R\right)\left[{f}_{\mathrm{R}}\left(E,{V}_R\right)-{f}_L\left(E,{V}_L\right)\right] dE $$

**Table 1:** Device parameters following ITRS and IRDS requirement

Channel length	EOT (nm)	V_DS_ (V)	Node
7 nm	0.5	0.68	2019
5 nm	0.41	0.64	2021
3 nm	0.41	0.64	2024

Where,*V*_Bias_ is the bias and can be achieved by: *V*_Bias_  =  *V*_*R*_ ‐ *V*_*L*_, *T*(*E*, *V*_*L*_, *V*_*R*_) is the transmission coefficient of carriers, *f*_R_(*E*, *V*_*R*_) and *f*_*L*_(*E*, *V*_*L*_) are the Fermi-Dirac distribution function for cathode (drain) and anode (source), respectively.

## Results and discussion

**Fig. 4:**
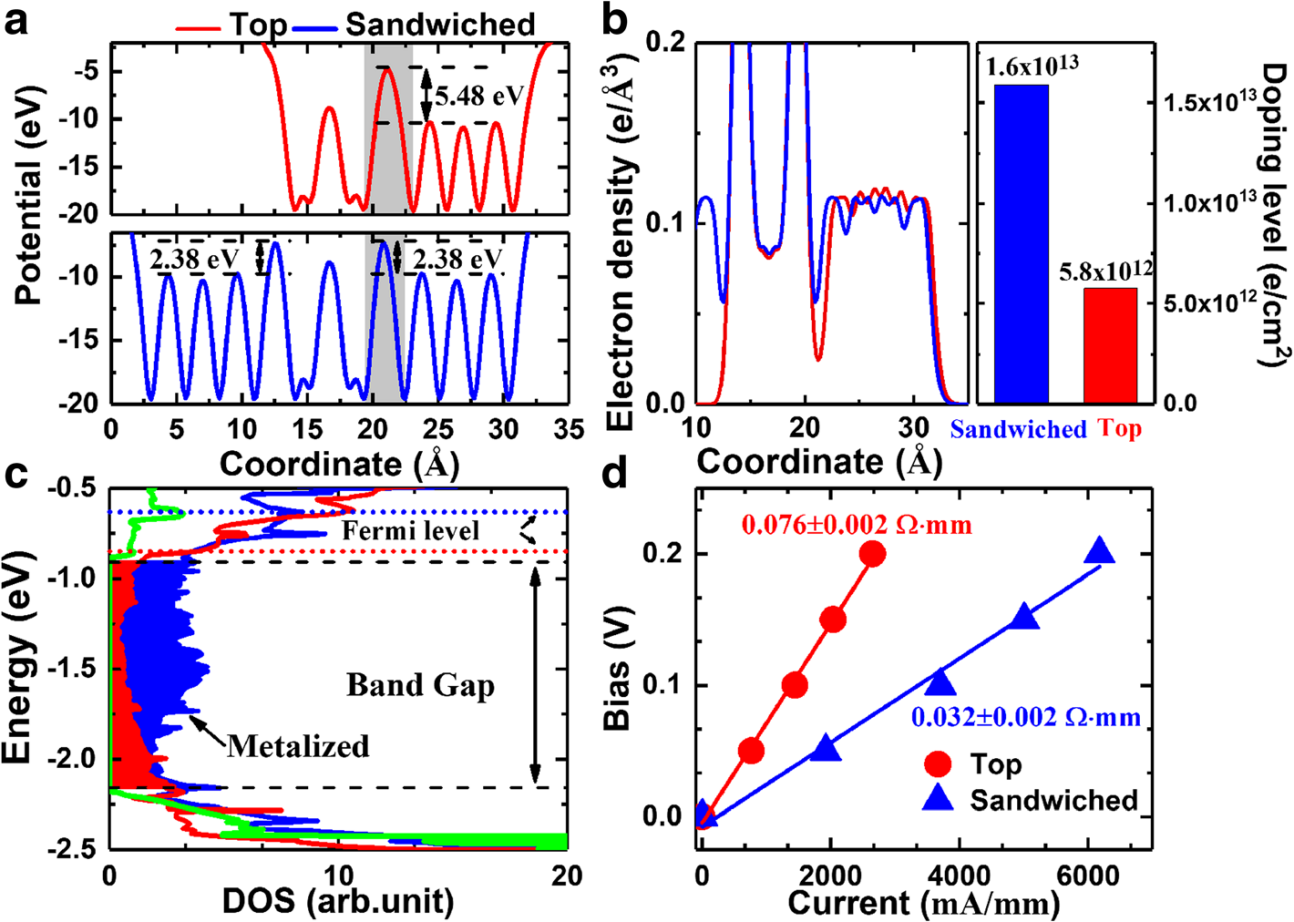
**a** Effective potential normal to the transport direction. The coordinate corresponds to the location of atoms and is defined in Fig. 1. The dark regions correspond to the vdW gap. **b** Plane-averaged electron distribution normal to the transport direction. The right panel is the doping level. The coordinate corresponds to the location of atoms and is defined in Fig. 1. **c** DOS of InSe. The green corresponds to pristine InSe. **d** Current dependent bias of the two probe devices. All of the red and blue correspond to top and sandwiched contacts, respectively

In general, there are three key factors correlated to the ohmic contact quality in 2D materials [18], i.e., the tunneling barrier and distance which is derived from vdW gap, orbital overlap between electrode and semiconductor, and also the SB height. First, the tunneling barrier and distance were described by effective potential shown in Fig. 4a. Compared to the top contact, introduction of sandwiched contact not only provides an additional transport path at the bottom side but also gives rise to a decrease of tunneling barrier from 5.48 to 2.38 eV, leading a reduction of 56.6%. Meanwhile, the interfacial distance also gets slightly lowered with 0.66 Å, denoting the width of tunneling barrier gets also reduced. Second, the orbital overlap can be evaluated from valance charge distribution in Fig. 4b. It can be noticed that sandwiched contact possesses more valance electrons at the interfacial region as compared to the top contact, indicating stronger orbital overlapping between indium and InSe. This feature also helps to introduce doping effect into InSe, and the excess electrons number can be calculated by using Mulliken population. We extracted the total number of electrons in InSe of sandwiched and top contacted structures, respectively. Then the doping level can be obtained by dividing the electron number by the area of the contact region, as net charge of isolated InSe should always be zero. As shown in the right panel of Fig. 4b, sandwiched contact yields a very high doping level of 1.6 × 10^13^ e/cm^2^, which is nearly 2.8 times higher than that of the top contact. Such a high level has approached the hypothesis in simulations of 2D tunneling FET, which usually claims much heavier doping level than metal-oxide-semiconductor FET. Thirdly, the density of states (DOS) of InSe in pristine, sandwiched, and top contacted structures are shown Fig. 4c. Orbital overlapping between indium and InSe at the interfacial region metallized the band gap of InSe, and sandwiched one results in a higher level. This feature greatly enhances carrier injection through vdW tunneling barrier at the interfacial region, as the metalized states in the band gap offer additional tunneling channels. In addition, the Fermi levels are pined above the conduction band minimum, resulting in energy degeneracy of ~ 0.07 and 0.27 eV for top and sandwiched contacts, respectively. Therefore, the SB between indium and InSe are completely eliminated. Fourthly, the ohmic contact resistance was calculated based on bias-current curve obtained from the two-probe devices, and all results are shown in Fig. 4d. We can notice both of contacts are ohmic due to the linear evolutions. At theoretical level, i.e., neglecting of surface roughness, interfacial impurities, etc., the sandwiched structure leads to a very low contact value of 0.032 ± 0.002 Ω mm, which reduces more than half of resistance of the top contact. Based on above discussions, it is interesting to notice that double the contact region always leads to more than twice improvement of the ohmic contact. Because top contact with indium was recently experimental confirmed to be effective to boost InSe-based devices performance [21, 22], sandwiched structure can be an appealing ohmic contact solution for InSe FETs.

**Fig. 5:**
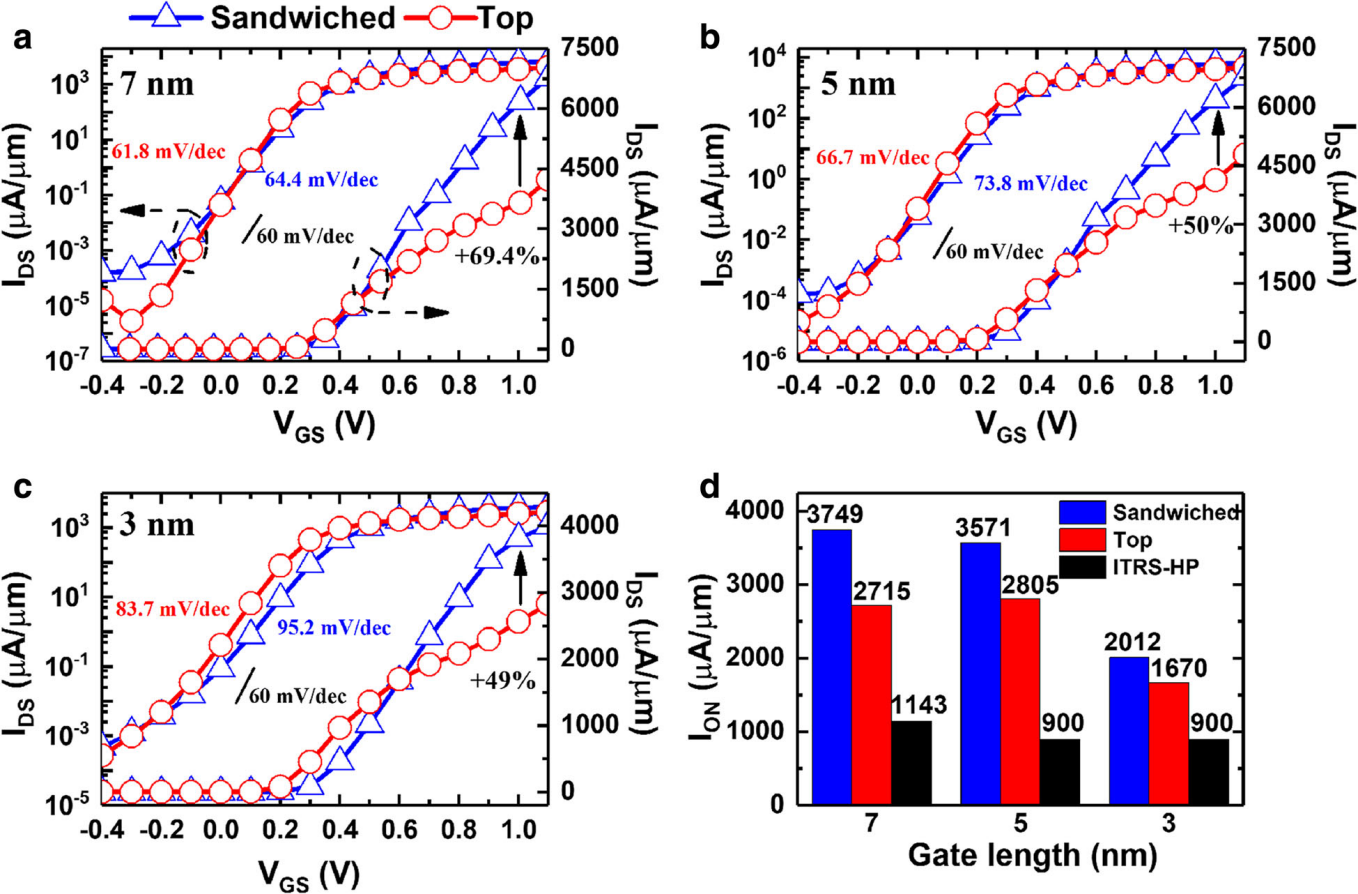
Transfer characteristics of InSe FETs at node. **a** 2019, **b** 2021, and **c** 2024 nodes, respectively. **d** Comparisons of I_ON_ following HP requirement of ITRS

Then, the device performance was evaluated, and the transfer characteristics of InSe FET at 2019, 2021, and 2024 nodes were shown in Fig. 5. It can be observed that the subthreshold swing (SS) of all nodes are below 100 mV/dec, and SS at 2019 node shows nearly ideal switching characteristics of 61.8 and 64.4 mV/dec for top and sandwiched contacted devices, respectively, indicating outstanding electrostatic control in InSe FETs. In addition, sandwiched contacted devices lead to evident improvement of I_DS_ compared to top ones with maximum increasement of 69.4%, 50%, and 49% being achieved at 2019, 2021, and 2024 nodes, respectively. Furthermore, I_ON_ was extracted following the requirement of high performance (HP) in the ITRS. As shown in Fig. 5d, I_ON_ of all systems is far above the HP requirement. Compared to top contacted devices, sandwiched systems still present a promotion of 38.2%, 27.3%, and 20.5% for 2019, 2021, and 2024 nodes, respectively.

**Fig. 6:**
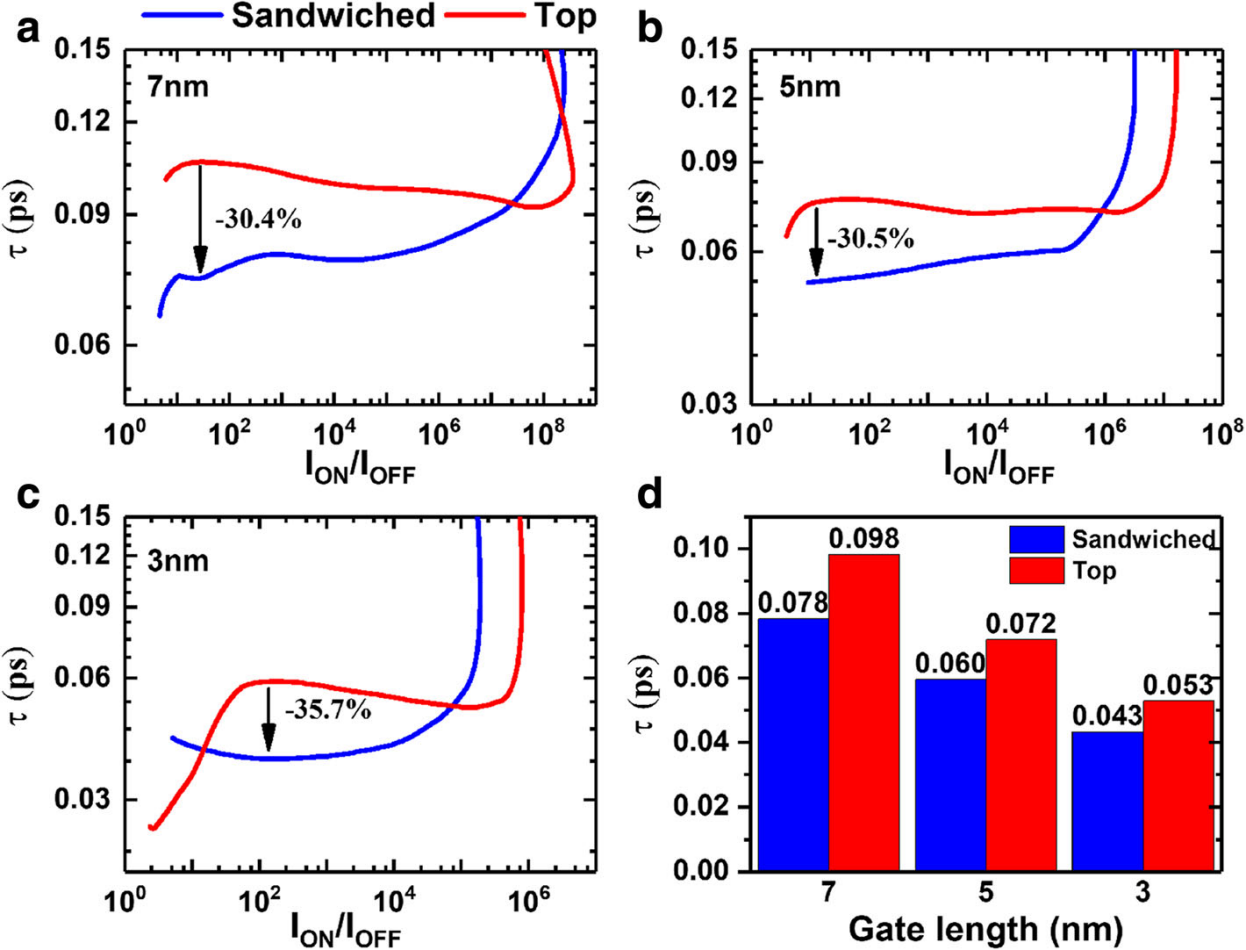
Intrinsic delay as a function of on-off ratio at node. **a** 2019, **b** 2021, and **c** 2024 nodes, respectively. **d** Comparisons of intrinsic delay following HP requirement of ITRS

Another essential metric of FET is intrinsic delay (τ), which signifies the upper limit of switching speed in the logical circuit. The τ was obtained by *τ* = (Q_ON_ − Q_OFF_)/I_ON_, where Q_ON_ and Q_OFF_ are charges at on and off states, respectively. The on and off states are constrained at |V_DS_|=0.68, 0.64, and 0.64 V for 2019, 2021, and 2024 nodes, respectively. Intrinsic delay as a function of on-off ratio is shown in Fig. 6. Despite the non-monotonic evolution at large delay which is derived from the tunneling under low gate voltages [41], all delays are below 0.15 ps and sufficiently lowered than the ITRS requirement of 0.44-0.46 ps. In addition, sandwiched contacted devices give rise to a reduction of more than 30% at regions of I_ON_/I_OFF_ ≤ 10^7^, 10^6^, 10^5^ for 2019, 2021, and 2024 nodes, respectively. On the basis of HP requirements shown in Fig. 6d, sandwiched contacted devices can still promote the switching speed with 20.4%, 16.7%, and 18.9% for 2019, 2021, and 2024 nodes, respectively.

**Fig. 7:**
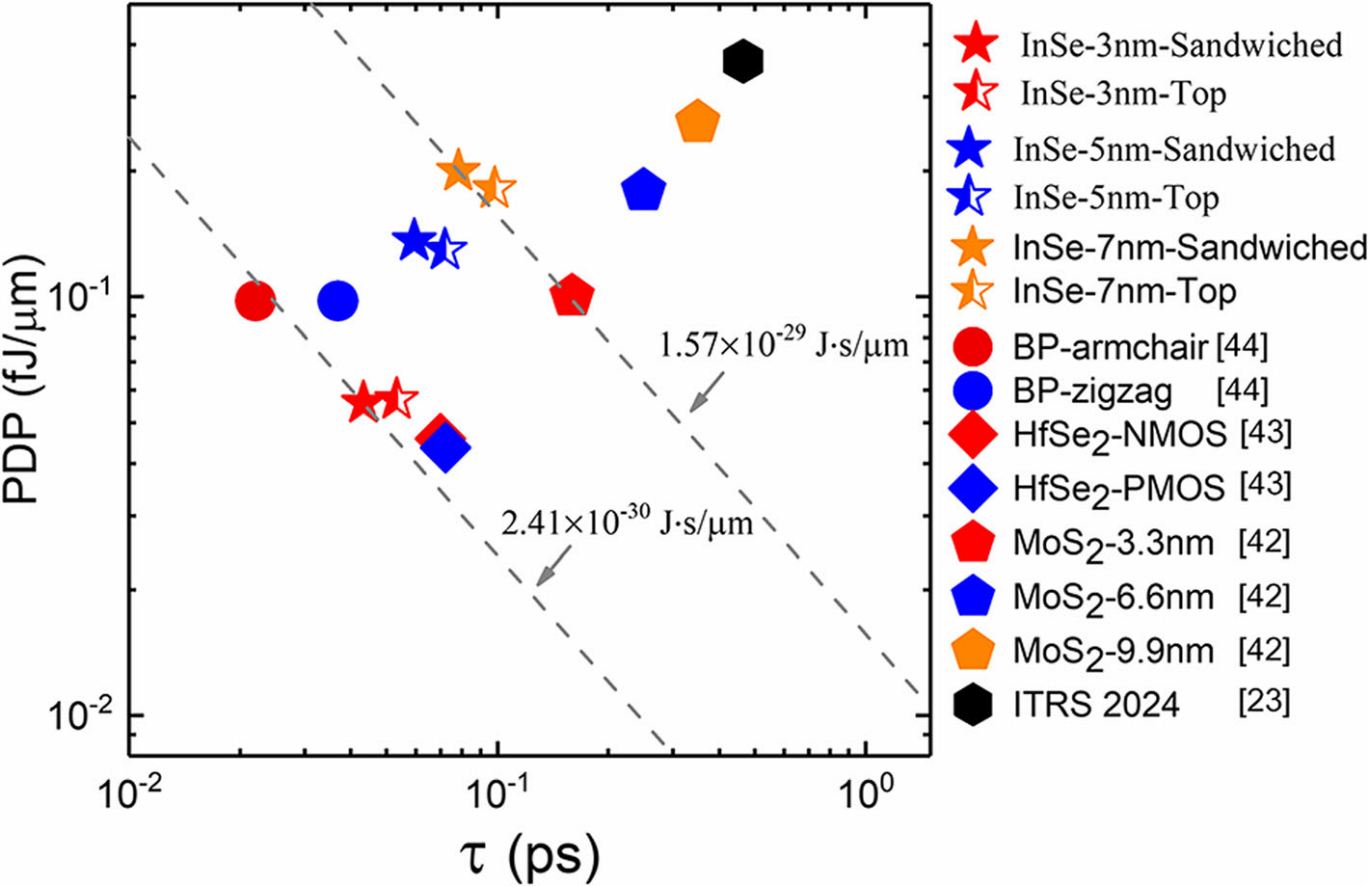
Power-delay product versus intrinsic delay comprised of InSe and other 2D FETs. The gray dashed guidelines correspond to specific EDP

In order to evaluate the device performance more intuitionally, power-delay product (PDP) versus intrinsic delay is extracted. PDP corresponds to the power consumption in a single switching event and is defined by PDP  =  (Q_ON_ − Q_OFF_)V_DS_ with all parameters derived from HP requirement of ITRS. Results and comparison with other 2D FETs are shown in Fig. 7. Firstly, all 2D FETs were selected based on the rule that they have been preliminarily verified as transistors in experimental reports, which goes a step further for CMOS technology. Secondly, except for InSe and MoS_2_ [42], all other devices were simulated with heavily doping in active regions and neglect of ohmic contact resistance [43, 44], therefore the results correspond to the upper limit of performance. As can be seen, all energy-delay product (EDP) are below ITRS 2024 requirement, indicating the appealing future of 2D FETs. The maximum of EDP belongs to MoS_2_ FET at 9.9 nm, and the best is from BP FET. As for InSe FETs, sandwiched contacted devices always perform better than top contacted ones at all nodes. The highest EDP of sandwiched contacted devices is at gate length of 7 nm (2019 node) and exceeds all MoS_2_ FETs. The lowest one is at gate length of 3 nm (2024 node) and even approaches the upper limit of BP FET in the armchair direction, which is well known for the outstanding transport properties. Accordingly, the EDP of InSe FET signifies that sandwiched contacted devices exhibit sufficient competitiveness among 2D FETs.

## Conclusions

In this work, a new sandwiched ohmic contact with indium was proposed for InSe FET. The sandwiched ohmic contact not only doubles the contact region but also promotes the contact quality more than twice, leading to an excellent contact resistance. At device performance level of gate length 7, 5, and 3 nm, InSe FETs with sandwiched ohmic contact present universal performance promotion as compared to conventional top contacted devices. Under the requirement of HP from ITRS, on-state current and intrinsic delay are improved with 38.2~20.5% and 20.4~16.7%, respectively. A benchmark of EDP against other 2D FETs also reveals that InSe FETs with sandwiched ohmic contact have advantages over other 2D FETs. Our study offers a new route toward high-performance InSe FETs.

## Data Availability

The datasets used and/or analysed during the current study are available from the corresponding author on reasonable request.

## Abbreviations

2D: Two-dimensional
CMOS: Complementary metal-oxide semiconductor
TMDs: Transition metal dichalcogenides
BP: Black phosphorus
InSe: Indium selenide
FET: Field-effect transistors
SB: Schottky barrier
2D FET: 2D materials-based FET
ITRS: International Technology Roadmap for Semiconductors
vdW: van der Waals
UL: Underlap
NEGF: Non-equilibrium Green’s function
DOS: The density of states
SS: Subthreshold swing
HP: High performance
τ: Intrinsic delay
PDP: Power-delay product
EDP: Energy-delay product
